# Methods for network meta-analysis of continuous outcomes using individual patient data: a case study in acupuncture for chronic pain

**DOI:** 10.1186/s12874-016-0224-1

**Published:** 2016-10-06

**Authors:** Pedro Saramago, Beth Woods, Helen Weatherly, Andrea Manca, Mark Sculpher, Kamran Khan, Andrew J. Vickers, Hugh MacPherson

**Affiliations:** 1 Centre for Health Economics, University of York, York, UK; 2 Warwick Clinical Trials Unit, Division of Health Sciences, Warwick Medical School, University of Warwick, Coventry, UK; 3 Department of Epidemiology and Biostatistics, Memorial Sloan-Kettering Cancer Center, New York, USA; 4 Department of Health Sciences, University of York, York, UK

**Keywords:** Evidence synthesis, Network meta-analysis, Mixed treatment comparisons, Individual-patient data, Analysis of covariance, Continuous outcome, Heterogeneity

## Abstract

**Background:**

Network meta-analysis methods, which are an extension of the standard pair-wise synthesis framework, allow for the simultaneous comparison of multiple interventions and consideration of the entire body of evidence in a single statistical model. There are well-established advantages to using individual patient data to perform network meta-analysis and methods for network meta-analysis of individual patient data have already been developed for dichotomous and time-to-event data. This paper describes appropriate methods for the network meta-analysis of individual patient data on continuous outcomes.

**Methods:**

This paper introduces and describes network meta-analysis of individual patient data models for continuous outcomes using the analysis of covariance framework. Comparisons are made between this approach and change score and final score only approaches, which are frequently used and have been proposed in the methodological literature. A motivating example on the effectiveness of acupuncture for chronic pain is used to demonstrate the methods. Individual patient data on 28 randomised controlled trials were synthesised. Consistency of endpoints across the evidence base was obtained through standardisation and mapping exercises.

**Results:**

Individual patient data availability avoided the use of non-baseline-adjusted models, allowing instead for analysis of covariance models to be applied and thus improving the precision of treatment effect estimates while adjusting for baseline imbalance.

**Conclusions:**

The network meta-analysis of individual patient data using the analysis of covariance approach is advocated to be the most appropriate modelling approach for network meta-analysis of continuous outcomes, particularly in the presence of baseline imbalance. Further methods developments are required to address the challenge of analysing aggregate level data in the presence of baseline imbalance.

**Electronic supplementary material:**

The online version of this article (doi:10.1186/s12874-016-0224-1) contains supplementary material, which is available to authorized users.

## Background

Evidence synthesis tools are increasingly used to pool estimates of treatment effects from multiple randomised controlled trials (RCTs) to inform assessments of comparative effectiveness generally, and particularly in the context of health technology assessment. One such tool is network meta-analysis (NMA, also known as mixed treatment comparisons), which extends standard pair-wise meta-analysis by allowing the simultaneous synthesis of evidence on multiple treatments [1–4]. Most published work focuses on the pooling of aggregate outcome data (AD), but with the increasing availability of individual patient data (IPD) synthesis methods have recently emerged to utilise IPD [5–10]. The use of IPD allows the consistent use of statistical methods across the body of evidence. It also creates added value by offering the potential to reduce and/or explain network heterogeneity, tackle existing evidence inconsistencies [11], and to examine subgroup effects in patients where interventions might have an effectiveness profile which differs from that of the wider population [10, 12]. Despite its advantages, only a few methodological studies on the synthesis of IPD in NMA are available in the published literature and even fewer examples of its use within cost-effectiveness (CE) analysis exist [13]. Methods for NMA of IPD have focused mainly on a subset of the available types of outcomes, i.e. binary and time-to-event outcomes [10, 14]. Few publications exist dedicated to continuous outcomes [15, 16], an important outcome set in medical applications, as well as in complementary medicine and beyond. Recent publications by Hong et al. [15] and Thom et al. [16] explored and discussed the synthesis of continuous endpoints using IPD in NMA. While the former proposes a framework to pool multiple continuous outcomes under contrast- and arm-based parameterisations, the latter focused mainly on modelling observational evidence available in both IPD and AD formats. Both papers chose the change from baseline as their continuous outcome for synthesis but did not adjust for baseline values of the outcome, apart from when modelling baseline outcome as a treatment-effect modifier [15]. In this paper we present a model for NMA of IPD on continuous outcomes using the analysis of covariance (ANCOVA) approach which does adjust for baseline outcome data.

Analysis of covariance (ANCOVA), where the outcome at follow-up is modelled whilst adjusting for its baseline value, is the preferred method for estimating treatment effects from continuous outcomes [17–19]. Treatment effect estimates based on ANCOVA methods are the most precise estimates and are robust to chance baseline imbalance. As such, these should be the desired outcome measure for synthesis [20–22]. Unfortunately, ANCOVA results are frequently not reported for individual studies and, therefore, ANCOVA is often not used in the synthesis of aggregate evidence. Instead, sub-optimal methods are used [23–25] such as unadjusted differences in change from baseline or final outcome measures.

When IPD is available from each study, the full set of statistical approaches are available to analysts. Riley et al. [22] discuss different approaches to the synthesis of continuous outcome data when IPD is available in a pair-wise meta-analysis framework. The authors highlight that availability of IPD is crucial to implement the most appropriate modelling approach, the ANCOVA [19, 22]. To our knowledge, such an ANCOVA synthesis model has not yet been extended to and/or explored in the NMA setting.

In this paper we present a Bayesian NMA model for the synthesis of continuous IPD using the ANCOVA framework. The paper aims to ensure that best practice in the analysis of continuous outcome data within individual trials and pairwise meta-analyses is extended to the NMA context. We also aim to illustrate the differences (and similarities) between NMA of IPD when using ANCOVA, change score and final score only approaches. The method presented is applied to a case study of acupuncture for chronic pain. The paper is structured as follows. Section 2 presents the motivating example for the manuscript, describes the evidence available and outlines the analysis undertaken to obtain outcome data for synthesis. Section 3 describes the NMA ANCOVA model for IPD on continuous outcomes, followed by extensions that incorporate treatment effect–covariate interactions. Results of applying the methods described to the motivating dataset are reported in Section 4, which is followed by some discussion topics and concluding remarks in Section 5.

### Evidence on the effectiveness of acupuncture for chronic pain in primary care

There is currently a lack of agreement about the effectiveness of acupuncture as a treatment for chronic pain, as reflected in debates about recent UK guidance surrounding its value [26–32]. Acupuncture received a positive recommendation from the National Institute for Health and Care Excellence (NICE) for its use in back pain [26] and headache/migraine [27], while a negative recommendation was given for its use in osteoarthritis in 2008 and 2014 [28]. The methods in this paper were developed as part of a project to improve evidence regarding the effectiveness and CE of acupuncture for chronic non-specific pain to inform decision making in the UK National Health Service [33].

#### Dataset

The data used in this study was provided by the Acupuncture Trialists’ Collaboration (ATC) who performed a systematic review in which relevant high quality trials were identified and, for a large proportion of trials, IPD was obtained (29 out of 31 studies) [34, 35]. The dataset available to us comprised 28 out of these 29 RCTs which assessed the effectiveness of acupuncture in three pain conditions: osteoarthritis of the knee (OAK) (7 trials [36–42]), headache, including tension-type headache (TTH) and migraine (6 trials [43–48]) and musculoskeletal conditions, encompassing lower back, shoulder and neck pain (15 trials [49–63]). This dataset comprises 17,512 patients. These studies are summarised in Table 1.

**Table 1:** Main characteristics of the data and study outcomes used for analysis

ID	Study 1^st^ author, year	Location	Pain group (type)	Age – mean (SD)	Trial follow-up period / Time point used in the analysis (months)	Treatment	Obser-vations	HRQoL outcome mapped	Pain outcome standardised
1	Diener 2006 [46]	Germany	Headache (migraine)	37.62 (10.4)	6 / 3	Usual care	328	SF-12	Migraine days
						Sham acupuncture	202		
						Acupuncture	305		
2	Endres 2007 [47]	Germany	Headache (TTH)	38.44 (11.77)	6 / 3	Sham acupuncture	200	SF-12	TTH days
						Acupuncture	209		
3	Jena 2008 [48]	Germany	Headache (headache)	43.66 (12.69)	6 / 3	Usual care	1613	SF-36	Headache days
						Acupuncture	1569		
4	Linde 2005 [44]	Germany	Headache (migraine)	42.55 (11.35)	6 / 3	Usual care	76	SF-36	Days of moderate to severe pain
						Sham acupuncture	81		
						Acupuncture	145		
5	Melchart 2005 [45]	Germany	Headache (TTH)	42.68 (13.18)	6 / 3	Usual care	75	SF-36	Headache days
						Sham acupuncture	62		
						Acupuncture	132		
6	Vickers 2004 [43]	UK	Headache (headache)	46.34 (10.39)	12 / 3	Usual care	161	SF-36	Severity score
						Acupuncture	140		
7	Brinkhaus 2006 [55]	Germany	Musculoske-letal (low back)	58.81 (9.13)	12 / 2	Usual care	79	SF-36	VAS pain score
						Sham acupuncture	73		
						Acupuncture	146		
8	Carlsson 2001 [50]	Sweden	Musculoske-letal (low back)	49.84 (15.4)	6 / 3	Sham acupuncture	16	VAS pain	VAS pain score
						Acupuncture	34		
9	Guerra 2004 [53]	Spain	Musculoske-letal (shoulder)	59.19 (11.37)	6 / 3	Sham acupuncture	65	VAS pain	VAS pain score
						Acupuncture	65		
10	Haake 2007 [61]	Germany	Musculoske-letal (low back)	50.15 (14.68)	6 / 3	Usual care	388	SF-12	Von Korff pain intensity score
						Sham acupuncture	387		
						Acupuncture	387		
11	Irnich 2001 [51]	Germany	Musculoske-letal (neck)	NA	3 / 3	Sham acupuncture	61	VAS pain	VAS pain score
						Acupuncture	56		
12	Kennedy 2008 [62]	Northern Ireland	Musculoske-letal (low back)	45.58 (11.1)	3 / 3	Sham acupuncture	24	VAS pain	Roland Morris disability score
						Acupuncture	24		
13	Kerr 2003 [52]	Northern Ireland	Musculoske-letal (low back)	NA	6 / 1	Sham acupuncture	20	VAS pain	VAS pain score
						Acupuncture	26		
14	Kleinhenz 1999 [49]	Germany	Musculoske-letal (shoulder)	NA	3 / 1	Sham acupuncture	27	CMS and predicted VAS pain	CMS
						Acupuncture	25		
15	Salter 2006 [56]	UK	Musculoske-letal (neck)	47.71 (16.51)	3 / 3	Usual care	14	no mapping – EQ-5D available	Northwick Park pain score
						Acupuncture	10		
16	Thomas 2006 [58]	UK	Musculoske-letal (neck)	42.62 (10.71)	24 / 3	Usual care	80	no mapping – EQ-5D available	SF-36 bodily pain score
						Acupuncture	159		
17	Vas 2006 [57]	Spain	Musculoske-letal (neck)	46.73 (13.2)	6 / 1	Sham acupuncture	62	SF-36	VAS pain score
						Acupuncture	61		
18	Vas 2008 [63]	Spain	Musculoske-letal (shoulder)	55.68 (11.37)	12 / 3	Sham acupuncture	220	VAS pain	CMS
						Acupuncture	205		
19	White 2004 [54]	UK	Musculoske-letal (neck)	53.36 (15.61)	12 / 3	Sham acupuncture	65	VAS pain	VAS pain score
						Acupuncture	70		
20	Witt 2006 [59]	Germany	Musculoske-letal (neck)	50.57 (12.93)	6 / 3	Usual care	1698	SF-36	Neck pain and disability score
						Acupuncture	1753		
21	Witt 2006 [60]	Germany	Musculoske-letal (low back)	52.83 (13.33)	6 / 3	Usual care	1390	SF-36	Hanover functional ability score
						Acupuncture	1451		
22	Foster 2007 [41]	UK	Osteoarthritis of the knee	63.23 (8.81)	12 / 1	Usual care	116	VAS pain	WOMAC pain score
						Sham acupuncture	119		
						Acupuncture	117		
23	Berman 2004 [36]	USA	Osteoarthritis of the knee	65.46 (8.62)	6 / 2	Usual care	189	no mapping – EQ-5D available	WOMAC pain score
						Sham acupuncture	191		
						Acupuncture	190		
24	Scharf 2006 [39]	Germany	Osteoarthritis of the knee	62.81 (10.07)	6 / 3	Usual care	316	SF-12	WOMAC total score
						Sham acupuncture	365		
						Acupuncture	326		
25	Vas 2004 [37]	Spain	Osteoarthritis of the knee	67.04 (10.09)	3 / 3	Sham acupuncture	49	WOMAC total	WOMAC total score
						Acupuncture	48		
26	Williamson 2007 [42]	UK	Osteoarthritis of the knee	70.67 (8.94)	3 / 3	Usual care	61	WOMAC total	Oxford knee score
						Acupuncture	60		
27	Witt 2005 [38]	Germany	Osteoarthritis of the knee	64.01 (6.49)	12 / 2	Usual care	70	SF-36	WOMAC total score
						Sham acupuncture	75		
						Acupuncture	149		
28	Witt 2006 [40]	Germany	Osteoarthritis of the knee	61.2 (10.39)	6 / 3	Usual care	310	SF-36	WOMAC total score
						Acupuncture	322		

**Fig. 1:**
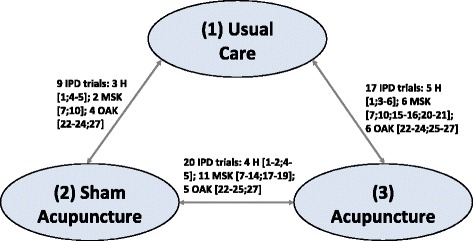
Network of RCTs. Legend: In the network, a unique treatment category is indicated by a circle. Arrows between circles indicate that these treatments have been compared in a trial (trials are identified using ‘[]’, numbered according to column ‘ID’ in Table 1. (Pain groups: H – Headache/migraine; MSK – Musculoskeletal; OAK – Osteoarthritis of the knee)

The dataset includes 11 trials comparing acupuncture to sham acupuncture, 8 comparing acupuncture and usual care, and 9 comparing all three comparators. The resulting evidence network is presented in Fig. 1.

#### Outcomes

One key aspect of the evidence available in this setting is the heterogeneous reporting of relevant outcomes across trials. The ATC dataset varied according to the type of outcomes reported but also on how these were collected across time. To address this issue, two outcome measures are used within this paper. The first is a standardised pain-related outcome, a dimensionless measure of treatment effect usually termed standardised mean difference (SMD) [20, 21, 64, 65]. For this analysis the primary outcome of each study was used to generate patient-level standardised pain estimates. Pain measures varied from days with headache in the headache/migraine pain condition, to visual analogue scale (VAS) pain in the musculoskeletal group or to Western Ontario and McMaster Universities Arthritis Index (WOMAC) pain in the OAK group, as reported in Table 1 (column on the right hand side). Individual-level standardised pain estimates were obtained for each trial by dividing the primary outcome scores by the study-specific standard deviation. Note that while these estimates were used as inputs in the synthesis models, the outputs of the synthesis are in the SMD format, as differences between treatments were estimated within the modelling^1^.

While SMDs may be useful for detecting differences between interventions, they are of limited value to decision making as these cannot directly inform estimates of absolute effect or CE modelling, unless they are first transformed [20]. These considerations motivated the second synthesis approach used, which involved translating (or ‘mapping’) the available patient-reported outcome data from the trials into EuroQol five-dimension (EQ-5D) index values [66]. The EQ-5D is a popular preference-based generic health-related quality of life (HRQoL) measure, typically employed to weight life years gained and thus derive quality-adjusted life-years (QALYs) for use in CE analysis [67]. The EQ-5D preference score was the second outcome explored. Due to its importance in supporting health system decision making processes, the EQ-5D preference score, used in CE analysis, has applications in many jurisdictions worldwide, including the UK [68]. The conventional EQ-5D questionnaire includes five domains, each of which can be at one of three severity levels. Using an algorithm, responses to this questionnaire can be transformed to a numeric value that reflects the preferences of the public for different heath states (here we used values from the UK general public [69]). Values range from −0.594 to 1 (the bounds represent, respectively, the worst imaginable health state and full health, with zero relating to death).

Only a small number of trials (*n* = 2) in the dataset directly provided EQ-5D data [36, 56]. Where such data were not available it was predicted using other generic and disease specific measures^2^ (Table 1) through published mapping algorithms. In 50 % (*n* = 14) of the trials, well established published algorithms were used to map from Short Form (SF)-36 dimensions and SF-12 summary scores to EQ-5D^3^ [58, 70, 71]. In 10 of the 28 trials, published algorithms which map VAS pain scores [72] and WOMAC scores [73] to EQ-5D were used^4^. For one trial, a double mapping approach was necessary as, to our knowledge, no direct mapping algorithm exists to obtain EQ-5D values from Constant Murley Score (CMS). Thus, an unpublished mapping algorithm (a report describing the derivation of the mapping algorithm is available on request from Kamran Khan: K.A.Khan@warwick.ac.uk [74]) was used to derive VAS pain estimates from CMS, which were then used to obtain individual-level EQ-5D predictions using the Maund et al. [72] algorithm.

For the majority of mapping models used, the proportion of total variation explained (quantified by the coefficient of determination, *R* ^*2*^, in most cases) was low. To account for this additional source of uncertainty, an additional variance component was included^5^ [75]. This was achieved by adding to each individual-level EQ-5D prediction a draw from a normal distribution with mean zero and variance equal to the study-specific residual variance, that is, $Var\left[\widehat{EQ5D}\right]\cdot \left(1-{R}^2\right)$, where $\widehat{EQ5D}$ is the predicted (mapped) EQ-5D at individual-level.

HRQoL and standardised pain estimates were obtained at baseline and at the follow-up period closest to 3 months following the start of treatment, as 3 months is the typical end of treatment measurement, though not necessarily the trial’s primary end-point. Changes from baseline were obtained by calculating the difference between values for these two time points. Missing data in the ATC dataset (9.3 % (*n* = 1,622) and 15.5 % (*n* = 2,716) of the total number of patients in the standardised pain and HRQoL outcome, respectively) was assumed to be missing at random (MAR) and a complete-case analysis was conducted.

Additional file 1: Table A1 presents the standardised pain outcome and (mapped/predicted) EQ-5D data. For both outcome measures baseline imbalance can be observed in some trials. The source of this imbalance is not clear, but should be addressed in the synthesis [76, 77].

## Methods

### Statistical models for the data

All analyses were conducted using Bayesian methods. A contrast-based modelling approach is taken throughout featuring relative treatment effects, in line with the parameterisation used by Lu and Ades [78], Saramago et al. [10] and others. A one-step modelling approach, where the likelihood for data at the IPD level and that of parameter estimates were described simultaneously, was preferred because we intended to explore treatment-by-covariate interactions at the patient-level [7, 79]. Note that all four models described below include pain type interactions which are specific to the current case-study. Table 2 summarises the key characteristics of the four models implemented, highlighting existing differences across these.

**Table 2:** Summary of key characteristics of implemented models

	Model 1	Model 2	Model 3	Model 4
Outcome type	Continuous	Continuous	Continuous	Continuous
Outcome synthesised	Change from baseline	Change from baseline	Change from baseline	Final score
Approach	ANCOVA (baseline adjustment)	ANCOVA (baseline adjustment)	No baseline adjustment	No baseline adjustment
Pain interactions (case-study specific)	Yes	Yes	Yes	Yes
Further adjustments	None	Patients characteristics as treatment-effect modifiers	None	None

#### ANCOVA analysis (model 1)

The main modelling approach considered (model 1) is a variation of the ANCOVA approach that models the change score adjusting for baseline outcome values and with no stratification variables [19, 22, 80, 81] – such an approach is seen as equivalent to the existing ANCOVA approach.

The model considers a set of *J* studies for which IPD was available. The set of treatments included in these trials are labelled [A,B,C], where A is the reference treatment and there are *K* (=3) treatments in total. At baseline, patient *i* in study *j* allocated to treatment *k* provides a baseline measurement *Y*_*ijk*0_ (where *0* indicates time *t* at baseline). Each patient provides a follow-up measurement (the assessment closest to 3 months), *Y*_*ijk*3_. The change from baseline (*Y*_*ijk*3_ − *Y*_*ijk*0_) is denoted *ΔY*_*ijk*_ and is assumed normally distributed with mean *θ*_*ijk*_ and study-level variance of *V*_*j*_.

*θ*_*ijk*_, is assumed to be a function of *μ*_*jb*_, the outcome for treatment *b* (the lowest indexed treatment in each study) in study *j* for a patient with a baseline utility of *0*, *Y*_*ijk*0_; *δ*_*jbk*_, the study-specific treatment effect for treatment *k* relative to treatment *b*; and *X*_*jp*_, *p - 1* dummy variables representing pain type *p* in the *j* ^*th*^ study. The latter terms were included to allow treatment effects to vary according to pain type (i.e. OAK; headache - including TTH and migraine; and musculoskeletal conditions - including lower back, shoulder and neck pain). There are different ways in which interaction effects can be specified in NMAs [82]. For this example we assumed that pain treatment interaction effects, *β*_*bkp*_, were different for each treatment but exchangeable across treatments. Estimates of *β*_*bkp*_ were therefore assumed to be drawn from a random distribution with a common mean (*B*_*p*_) and between treatment variance (*σ*_*Bp*_^2^). An exchangeable interaction approach for pain was thought to be the most appropriate as it allowed pain interactions to be different across treatments but related. Pain interaction effects were not included for OAK as this is used as the reference pain indication. Pain interaction terms were specific to the current application and may be excluded if not of interest. However, we emphasise that adjustment for baseline should always be included regardless of the need to model interactions.

A random treatment effect approach was taken due to the expected between-study heterogeneity, the variance of which is described as σ^2^.

This model can be written as:

1 $$ \begin{array}{l}\Delta {Y}_{ijk}\sim N\left({\theta}_{ijk},{V}_j\right)\\ {}{\theta}_{ijk}=\left\{\begin{array}{cc}\hfill {\mu}_{jb}+{\beta}_{0j}{Y}_{ijk0}\hfill & \hfill if\kern0.5em k=b;\kern0.5em b\in \left\{A,\kern0.5em B,\kern0.5em C,\dots \right\}\hfill \\ {}\hfill {\mu}_{jb}+{\beta}_{0j}{Y}_{ijk0}+{\delta}_{jbk}+{\beta}_{bkp}{X}_{jp}\hfill & \hfill if\kern0.5em k>b\hfill \end{array}\right.\\ {}{\delta}_{jbk}\sim N\left({d}_{bk},{\sigma}^2\right)\sim N\left({d}_{Ak}-{d}_{Ab},{\sigma}^2\right)\\ {}{\beta}_{bkp}={\beta}_{Akp}-{\beta}_{Abp}\\ {}{\beta}_{Akp}\sim N\left({B}_p,{\sigma}_{Bp}^2\right)\\ {}{d}_{AA},{\beta}_{AAp}=0\end{array} $$

Prior distributions were defined independently as follows: 1/*V*_*j*_ ∼ *Gamma* (0.001, 0.001); *μ*_*jb*_ ∼ *N*(0, 10^6^); *β*_*oj*_ ~ *N*(0, 10^6^); *d*_*Ak*_ ∼ *N*(0, 10^6^); *σ* ∼ *Unif*(0, 2); *B*_*p*_ ∼ *N*(0, 10^6^); *σ*_*Bp*_ ∼ *Unif*(0, 2). Correlations in the random effects from trials with three or more arms were accounted for using published methodology [3, 64]. In this paper, *k > b* indicates that *k* is after *b* in the alphabet.

#### Controlling for treatment effect modifying patient-level characteristics (model 2)

For the EQ-5D endpoint, model 1 was extended to include patient-level covariates as potential treatment effect modifiers. Clinical expectations were that older age or higher body max index (BMI) may make patients more difficult to treat and, thus, potentially reduce the effect of treatment. Data on age were available from most studies and it was included as a covariate (centred) in the synthesis model. Again, a range of approaches can be used to incorporate treatment-effect interactions. In this analysis we assumed a common effect across pain types and for both acupuncture and sham acupuncture (i.e. a single interaction term is assumed to apply to all comparisons with usual care) [82] as this was deemed more clinically plausible. A non-linear effect of age was expected *a priori*, and thus squared terms were included for both main effects and treatment interaction effects. BMI data were only available in 10 of the 28 studies and for this reason we did not explore this variable further.

Model 2 thus differs from model 1 in that it considers the effects of the covariate Z (age). Differences to model 1 are shown below:

2 $$ \begin{array}{l}{\theta}_{ijk}=\left\{\begin{array}{cc}\hfill {\mu}_{jb}+{\beta}_{0j}{Y}_{ijk0}+{\phi}_0{Z}_{ijk}+{\varphi}_0{Z}_{ijk}^2\hfill & \hfill if\ k=b;\ b\in \left\{A,B,C,\dots \right\}\hfill \\ {}\hfill {\mu}_{jb}+{\beta}_{0j}{Y}_{ijk0}+{\phi}_0{Z}_{ijk}+{\varphi}_0{Z}_{ijk}^2+{\delta}_{jbk}+{\beta}_{bkp}{X}_{jp}\hfill & \hfill if\ k>b\ \mathrm{and}\ b\ne A\hfill \\ {}\hfill {\mu}_{jb}+{\beta}_{0j}{Y}_{ijk0}+{\phi}_0{Z}_{ijk}+{\varphi}_0{Z}_{ijk}^2+\phi {Z}_{ijk}+\varphi {Z}_{ijk}^2+{\delta}_{jbk}+{\beta}_{bkp}{X}_{jp}\hfill & \hfill if\ k>b\ \mathrm{and}\ b=A\hfill \end{array}\right.\\ {}{Z}_{ijk}\sim N\left(m,{\sigma}_Z^2\right)\end{array} $$

Coefficients on the main covariate effect and the effect squared are represented by *ϕ*_0_ and *φ*_0_. Coefficients on the treatment-by-covariate interaction term and the interaction between treatment and the squared covariate term are represented by *ϕ* and *φ*. No interaction term for comparisons of *k* and *b* were included when *b ≠ A* because the common regression coefficient cancels out.

Due to the possibility of missing covariate information for some individuals in some studies, *Z*_*ijk*_ was assumed to be a normally distributed random variable with mean *m* and variance *σ*_*Z*_^2^, common across all IPD studies. This represents a Markov chain Monte Carlo (MCMC) multiple imputation technique which generates independent draws of the missing data from its predictive distribution assuming MAR covariate data. Additional priors were required for this model: *ϕ*_0_, *ϕ*, *φ*_0_, *φ* ∼ *N*(0, 10^6^); *m* ∼ *Unif*(−50, 50), *σ*_*z*_ ~ *Unif*(0, 30)

#### Analysis with restricted evidence (model 3 and 4)

Although model 1 is the preferred choice, this model would not be feasible in the absence of outcome information at the individual-level for both baseline and follow-up time points. Sub-optimal models which do not rely on the availability of IPD were therefore run for comparison purposes. Three options are typically available to the analyst when only AD are available [22] – i) in the event of ANCOVA estimates being available, synthesise these using published literature [22]; or ii) model the change score without baseline adjustment (model 3); or iii) model the final outcome score without baseline adjustment (model 4). We note that – though suboptimal - model 3 has also been presented in the context of an NMA of continuous outcomes when IPD were available [15, 16].

Models 3 and 4 are simplifications of model 1 where the baseline outcome variable is omitted and where model 4 considers the final score, rather than the change score, as dependent variable. The synthesis of data using models that ignore baseline outcomes may provide biased treatment effect estimates because of potential baseline imbalances (unless addressed within trials themselves) and due to ignoring potential correlation between the change/final score and the baseline value [77, 83]. It may also reduce the precision of treatment effect estimates, even if balance at baseline is observed across all synthesised evidence [22].

#### Calculating the residual deviance

The total residual deviance, *TRD* – a measure of model fit - can be estimated for each of the described models by summing study-level residual deviances, *RD*. Study-level *RDs* are the ratio of the sum across studies of the squared differences between the observed changes from baseline, *ΔY*_*ijk*_, and the estimated mean, *θ*_*ijk*_, divided by the study-level variance, *V*_*j*_ [84]:

3 $$ \begin{array}{l}{D}_{ijk}={\left(\varDelta {Y}_{ijk}-{\theta}_{ijk}\right)}^2\\ {}R{D}_j= sum\left({D}_j\right)/{V}_j\\ {}TRD= sum(RD)\end{array} $$

For a model that fits the data well, it is assumed that the contributions to the *RD* to have a chi-squared distribution with *N* degrees of freedom if a sum over *N* unconstrained data points is made. On this basis, it is expected that the posterior mean of the *TRD* should be close to the number of unconstrained data points if the model predictions are a good fit to the data [20, 84, 85].

### Model selection and implementation

Data management was performed in the freely available software package R version 3.0.0 (Copyright © 2013 The R Foundation for Statistical Computing [86]). The NMA analyses were undertaken in WinBUGs [87] version 1.4.3 (Copyright © 2008 Medical Research Council (UK) and Imperial College (UK)), linked to the R software through the packages R2WinBUGS [88] and CodaPkg [89]. Annotated code, sample data and initial values for model 1 are provided in the Additional file 2 to allow readers to adapt it for their own purposes.

In all models the MCMC Gibbs sampler was initially run for 10,000 iterations and these were discarded as ‘burn-in’. Models were run for a further 5,000 iterations, on which inferences were based. Chain convergence was checked using autocorrelation and Brooks-Gelman-Rubin diagram [90, 91] diagnostics. Goodness of fit was assessed using the deviance information criterion (DIC) and TRD [84]. Results are presented as EQ-5D preference scores and SMD treatment effect estimates (and associated 95 % credibility intervals, CrIs) and also using the probability of treatment being the ‘best’ treatment in terms of being the most clinically effective [4].

## Results

### ANCOVA analysis results (model 1)

**Fig. 2:**
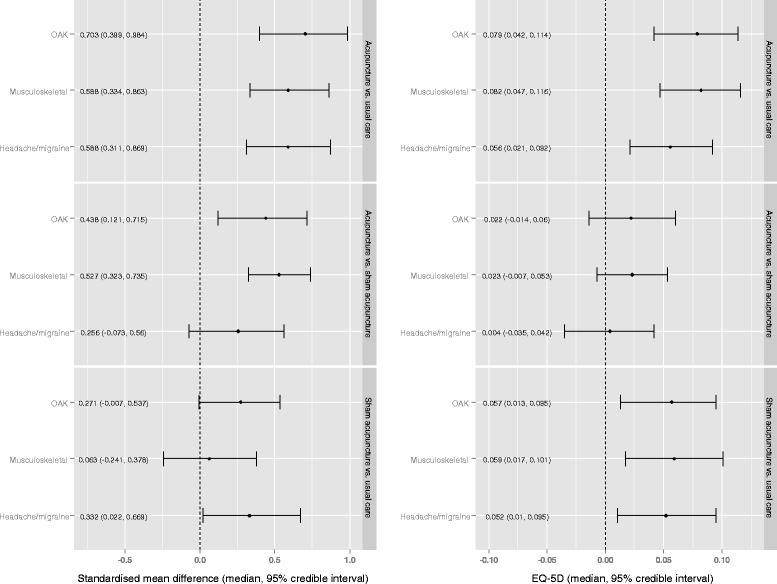
Forest plot showing network meta-analysis results for standardised pain and EQ-5D outcomes

Table 2 and Fig. 2 show the evidence from model 1 on relative treatment effect estimates adjusted for baseline and treatment-by-pain interaction effects (medians of the MCMC posterior samples and 95 % CrI shown). Measures of model fit (TRD and DIC) are also shown. The reference category for the pain interaction effects is the OAK pain type.

For both endpoints, model 1 indicates that acupuncture treatment increases the HRQoL of patients and/or reduces pain more than usual care and sham acupuncture treatments, irrespective of pain group. For the EQ-5D endpoint the treatment effect of acupuncture *vs.* usual care in the OAK population is 0.079 (median, 95 % CrI: 0.042 to 0.114), for headache/migraine and musculoskeletal pain patients the comparable treatment effects are 0.056 (median, 95 % CrI: 0.021 to 0.092) and 0.082 (median, 95 % CrI: 0.047 to 0.116), respectively. The results also favour acupuncture over sham acupuncture, although with a greater degree of uncertainty, as reflected by the fact that CrIs include zero for all pain types (OAK: 0.022, 95 % CrI −0.014 to 0.060; headache/migraine: 0.004, 95 % CrI −0.035 to 0.042; and musculoskeletal 0.023, 95 % CrI −0.008 to 0.053). The probability that acupuncture is the best treatment at improving HRQoL is 0.89 for OAK, 0.64 for headache/migraine and 0.95 for musculoskeletal pain.

Results for the SMD endpoint followed a similar pattern. However, in contrast to the EQ-5D analysis, in the latter comparison the CrIs do not include zero in the standardised pain analysis for OAK (0.438, 95 % CrI 0.121 to 0.715) and musculoskeletal (0.527, 95 % CrI 0.323 to 0.735) pain types, though they do for headache/migraine (0.256, 95 % CrI −0.073 to 0.560). The probability that acupuncture is the best treatment at improving standardised pain is 0.96 to 1.00 depending on pain type.

It is interesting to note that sham acupuncture *vs.* usual care treatment effect 95 % CrIs across pain types do not include *0* in the EQ-5D endpoint but they do for SMD, except for the headache/migraine group. These results suggest that sham acupuncture effects may well go beyond pain. Also interesting is the estimated magnitude of the uncertainty over the pain type interactions (not reported) as these, particularly for the EQ-5D endpoint, do not provide strong evidence of a difference between pain types.

Expectations were that some level of heterogeneity existed between-trials. Possibly as a consequence of the mapping work performed, this expectation was not fulfilled for the EQ-5D endpoint (the between-study variance estimate is 0.001). For the SMD endpoint the between study variance was also small relative to the magnitude of the treatment effects (the between-study variance estimate is 0.09). The TRD suggests that the models provide an adequate fit to the data (see Table 3).

**Table 3:** IPD NMA ANCOVA synthesis model results (model 1), EQ-5D preference score and standardised pain endpoints

IPD NMA ANCOVA results: EQ-5D preference score and SMD endpoints^a^			Model 1, ANCOVA, change in outcome score, adjusted for baseline median MCMC posterior sample (95 % CrI)	
			Change EQ-5D	Change standardised pain
Relative treatment effects	*Osteoarthritis of the knee*	*SHAM vs UC*	0.057 (0.013, 0.095)	0.271 (-0.007, 0.537)
		*ACU vs UC*	0.079 (0.042, 0.114)	0.703 (0.399, 0.984)
		*ACU vs SHAM*	0.022 (-0.014, 0.060)	0.438 (0.121, 0.715)
	*Headache*	*SHAM vs UC*	0.052 (0.010, 0.095)	0.332 (0.022, 0.669)
		*ACU vs UC*	0.056 (0.021, 0.092)	0.588 (0.311, 0.869)
		*ACU vs SHAM*	0.004 (-0.035, 0.042)	0.256 (-0.073, 0.560)
	*Musculoskeletal*	*SHAM vs UC*	0.059 (0.017, 0.101)	0.063 (-0.241, 0.378)
		*ACU vs UC*	0.082 (0.047, 0.116)	0.588 (0.334, 0.863)
		*ACU vs SHAM*	0.023 (-0.008, 0.053)	0.527 (0.323, 0.735)
	*Between-study variance*		0.001 (0, 0.003)	0.090 (0.049, 0.170)
	*Total residual deviance* ^*b*^		15,850 (15,480; 16, 230)	17,060 (16,660; 17,450)
	*Deviance information criterion* ^*c*^		-6,420.4	37,394.2

^a^ *UC* usual care, *SHAM* sham acupuncture, *ACU* acupuncture, *Headache group* headache, migraine and TTH, *Musculoskeletal group* neck, shoulder and low back pain

^b^For the EQ-5D endpoint models used approx. 14800 observations; for the SMD endpoint models used approx. 15900. Models should be preferred when total residual deviance mean posterior is close to the actual number of data points

^c^Deviance information criterion (DIC) is a statistical measure of model fit and model comparison. Models with smaller DIC are preferred

### Controlling for patient-level characteristics (model 2)

Table 1 provides information on age for each of the trials included in the dataset. The average age was lower in the headache/migraine pain group than in the musculoskeletal group, which in turn was lower than the OAK group.

Using the change in EQ-5D as the outcome for synthesis, Table 4 presents the results of applying model 2 (an extension of model 1) to include patient-level information on age – with age considered as a potential treatment effect modifier. The model fit statistics show that the adjusted by age model is marginally better than model 1, providing lower DIC statistics and reduced posterior RD. The results are very similar to model 1 and do not suggest age is a strong effect modifier or that non-linear effects of age on the effect of treatments are present.

**Table 4:** IPD NMA ANCOVA synthesis model (model 2) results with adjustments, EQ-5D preference endpoint

IPD NMA results: EQ-5D preference scores endpoint^a^			Model 2, ANCOVA, with adjustment for baseline score, age and treatment-by-age interactions, median MCMC posterior sample (95 % CrI)
Relative treatment effects	*Osteoarthritis of the knee*	*SHAM vs UC*	0.040 (-0.006, 0.084)
		*ACU vs UC*	0.066 (0.025, 0.105)
		*ACU vs SHAM*	0.026 (-0.012, 0.066)
	*Headache*	*SHAM vs UC*	0.056 (0.012, 0.098)
		*ACU vs UC*	0.060 (0.023, 0.095)
		*ACU vs SHAM*	0.004 (-0.036, 0.043)
	*Musculoskeletal*	*SHAM vs UC*	0.045 (-0.001, 0.094)
		*ACU vs UC*	0.074 (0.038, 0.109)
		*ACU vs SHAM*	0.029 (-0.009, 0.067)
Main effects	*Age*		-0.002 (-0.002, -0.001)
	*Age* ^*2*^		0.000 (0.000, 0.000)
Age common interactions	*Age*		0.000 (0.000, 0.001)
	*Age* ^*2*^		0.000 (0.000, 0.000)
	*Between-study variance*		0.001 (0.000,0.003)
	*Total residual deviance* ^*b*^		15,590 (15,210; 15,970)
	*Deviance information criterion* ^*c*^		-6,462.0

^a^ *UC* usual care, *SHAM* sham acupuncture, *ACU* acupuncture, *Headache group* headache, migraine and TTH, *Musculoskeletal group* neck, shoulder and low back pain

^b^Compare to approx. 14, 800 observations

^c^Deviance information criterion (DIC) is a statistical measure of model fit and model comparison. Models should be preferred with smaller DIC

### Analysis with restricted evidence (model 3 and 4)

Models 3 and 4 model the change score and the final outcome score, respectively. These are seen as simplifications of model 1 where no baseline adjustment is done. Results for models 3 and 4 are presented in Table 5, together with model 1 results for comparison. Generally, all three models convey the same message in relation to which treatment provides higher increases in patients’ HRQoL; that is, acupuncture is found to be better than sham and usual care treatments. As expected, models 3 and 4 (model 3 in particular) provide different summary results of treatment effects when compared to model 1. Compared with the ANCOVA model (model 1), model 3, the change score approach, generally inflates the summary treatment effects across pain types, with potential losses in precision (e.g. for OAK the median EQ-5D treatment effect is inflated 19 % in model 3 compared to model 1 for the acupuncture *vs* usual care comparison). Compared to model 1, model 4 summary treatment effects are generally similar or lower; CrIs are however consistently wider in model 4 compared to model 1.

**Table 5:** IPD NMA results for models (1), (3) and (4), EQ-5D preference score endpoint

IPD NMA results: EQ-5D preference scores endpoint^*a*^			Model 1, ANCOVA, change in EQ-5D scores, adjusted for baseline	Model 3, change in EQ-5D scores, without baseline adjustment	Model 4, follow-up EQ-5D score, without baseline adjustment
			Median MCMC posterior sample (95 % CrI)	Median MCMC posterior sample (95 % CrI)	Median MCMC posterior sample (95 % CrI)
Relative treatment effects	*Osteoarthritis of the knee*	*SHAM vs UC*	0.057 (0.013, 0.095)	0.077 (0.033, 0.118)	0.051 (0.008, 0.094)
		*ACU vs UC*	0.079 (0.042, 0.114)	0.093 (0.054, 0.129)	0.074 (0.035, 0.113)
		*ACU vs SHAM*	0.022 (-0.014, 0.060)	0.016 (-0.022, 0.054)	0.023 (-0.014, 0.065)
	*Headache*	*SHAM vs UC*	0.052 (0.010, 0.095)	0.044 (0.002, 0.086)	0.052 (0.007, 0.098)
		*ACU vs UC*	0.056 (0.021, 0.092)	0.057 (0.023, 0.090)	0.054 (0.016, 0.092)
		*ACU vs SHAM*	0.004 (-0.035, 0.042)	0.013 (-0.025, 0.051)	0.002 (-0.038, 0.040)
	*Musculoskeletal*	*SHAM vs UC*	0.059 (0.017, 0.101)	0.062 (0.019, 0.104)	0.054 (0.010, 0.099)
		*ACU vs UC*	0.082 (0.047, 0.116)	0.084 (0.048, 0.119)	0.080 (0.044, 0.118)
		*ACU vs SHAM*	0.023 (-0.008, 0.053)	0.022 (-0.011, 0.055)	0.026 (-0.006, 0.056)
	*Between-study variance*		0.001 (0, 0.003)	0.001 (0, 0.003)	0.001 (0, 0.003)
	*Total residual deviance* ^*b*^		15,850 (15,480; 16,230)	16,990 (16,570; 17,420)	15,370 (15,010; 15,730)
	*Deviance information criterion* ^*c*^		-6,420.4	-69.9	-3,823.7

^a^ *UC* usual care, *SHAM* sham acupuncture, *ACU* acupuncture, *Headache group* headache, migraine and TTH, *Musculoskeletal group* neck, shoulder and low back pain, *OAK* osteoarthritis of the knee

^b^Compare to approx. 14,800 observations

^c^Deviance information criterion (DIC) is a statistical measure of model fit and model comparison. Models should be preferred with smaller DIC

## Discussion

This study presents methods for conducting NMA of IPD on continuous outcomes, building on previous work on ANCOVA models for pairwise meta-analysis [22]. IPD availability avoided the use of non-baseline-adjusted models, allowing for ANCOVA models to be applied, thus improving precision of treatment effect estimates while adjusting for baseline imbalance [22]. Our results generalise the findings from Riley et al. [22] to the NMA setting and reinforce the idea that different approaches to the synthesis of continuous outcomes will produce different results. The ANCOVA approach is advocated to be the most appropriate modelling approach. Due to limited reporting of ANCOVA results in trial publications, IPD will typically be required to facilitate implementation of the ANCOVA NMA approach. The appropriate analysis of continuous endpoints therefore provides a further rationale for obtaining access to IPD, in addition to those well documented in the NMA literature [10, 12, 15, 92].

Recent work by Hong et al. [15] and Thom et al. [16] presented and discussed IPD NMA models for continuous outcomes. While Hong and colleagues [15] introduced contrast-based and arm-based models for multiple outcomes, Thom et al. [16] synthesised AD and IPD, some of which was observational rather than RCT data. They also considered interactions between treatment effects and covariates. The existence of ecological bias was explored in Hong et al. [15] by partitioning within- and across-study interactions [10]. Both publications used the change from baseline as their continuous outcome measure. In both publications models were presented that did not incorporate an adjustment for baseline outcome values, and in Hong et al. [15] adjustment for baseline outcome values was only considered in the context of modelling baseline outcomes as a treatment effect modifier. Thom et al. [16] recognised that the approach taken was not the recommended one, but noted that an ANCOVA-type approach was not possible as, for most studies in their motivating example, only AD was accessible to them. Our work emphasises that where IPD is available, all models of continuous outcomes should include adjustment for the baseline outcome, and unadjusted models should not be presented.

Analyses in this paper were conducted to explore the implications of using non-ANCOVA models in a NMA framework, as other methods have been used in the literature [15] to analyse continuous outcome IPD, and these methods are often necessary in the absence of IPD. The results showed some differences with the ANCOVA results. Modelling final scores or change scores without baseline adjustment produced estimates of treatment effect which differed by up to 19 % compared to the baseline adjusted model. By explicitly accounting for correlation between the change score and the baseline score in the presence of baseline imbalance, the tested ANCOVA model (model 1) avoids bias in the pooled treatment effect estimates. These results emphasise how important it is to adjust for baseline to adequately synthesise evidence in this setting; tasks very much facilitated with the availability of IPD. We hope that by highlighting the consequence of using suboptimal model(s) may encourage readers to obtain IPD so that the most appropriate methods may be implemented. When IPD is available ANCOVA should always be used. There has been a discussion in the literature about the fact that final or change score analyses may ‘bound’ the true relative effect estimate. Although this may be true for a single trial, it may not hold for NMA models [18]. This emphasises the importance of conducting appropriate analyses as the potential direction of bias is difficult to predict. Any bias in treatment effect or impact on precision could lead to inappropriate decisions regarding adoption and further research.

The motivating example related to the effectiveness of acupuncture for the treatment of chronic pain. The analyses found acupuncture to be more effective than usual care with respect to reducing pain and improving EQ-5D preference scores in patients with chronic pain of OAK, musculoskeletal and headache/migraine origin. The benefits of acupuncture over sham acupuncture are smaller than when compared to usual care. The methods used provided outputs in a format that can be used to directly inform CE considerations once the full set of relevant comparators are considered.

A recent study by Vickers et al. [35] also explored the effectiveness of acupuncture for chronic pain. This study performed an IPD pair-wise meta-analysis using the same data plus data from an additional trial [93] – data which, due to lack of consent, was not available to be used in the current analysis. Using study-specific primary outcome measures and the ANCOVA methodology, the Vickers et al. [35] study conducted meta-analyses separately for comparisons of acupuncture with sham acupuncture and usual care, and within each pain type. Despite the methodological differences, and differences for some trials in choice of primary outcome measure and/or primary end point, the authors’ findings are similar.

The instruments used to measure health outcomes differed between trials. Standardisation and mapping approaches were used to derive, pain-related outcomes and EQ-5D, respectively. Analysis of the pain outcome required development of methods for conducting standardised mean difference analysis with IPD. Analysis of the EQ-5D data required an extensive mapping exercise whereby separate mapping functions were applied to each study, with choice of mapping dependent on the available outcome data. Access to IPD in this context also avoided the use of any assumptions regarding the distribution of HRQoL instrument scores – thus allowing the observed distributions to be adequately reflected in the mapped EQ-5D estimates.

This study has a number of limitations. The applicability of these methods is conditional on the access to IPD. If IPD is not available or is partially available, other methods need to be used and limitations stressed. Often a mixture of IPD and AD is available – anecdotally a 50 % success rate of obtaining IPD is attained in the academic world, lower success rates may be achieved elsewhere, where it is common to have, for instance, only a company’s own RCT data and not that for competitor interventions. In the context of continuous outcomes the advantages of access to IPD are significant and efforts to share data should be pursued. As access to IPD for all studies in all NMAs is likely to be unrealistic in the medium-term, it would be useful to have available a methodology which had the advantages of the ANCOVA approach but could be used when only some (or even no) studies in the database were available in IPD form.

Additionally there are a series of limitations related to the case study. Firstly, the synthesis of heterogeneous outcomes relied on imperfect standardisation processes (which assume that any differences in within trial outcome variability are due to the use of different instruments) and mappings which are typically able to explain only a minority of variation in EQ-5D. The availability of key outcomes across trials would have reduced these concerns, as would the collection of generic preference based measures of HRQoL in all trials. Also, the outcome data closest to 3 months were selected for synthesis. For some trials, the nearest reported outcome data were at only months 1 or 2. If the effect of acupuncture increases gradually, these effects may underestimate 3 month outcomes. Furthermore, some of the trials show increased benefits of acupuncture over comparators at 12 [94] and 24 months [95] compared to 3 months. This evidence may be an indication of the long-term clinical benefits of acupuncture and has implications for estimating long-term HRQoL and CE. Collection of trial data for more than 3 months is therefore warranted together with further work analysing repeated outcome measurements in a NMA to evaluate the importance of these effects.

A complete-case analysis was conducted. This approach to missing data has been thoroughly documented in the methods literature as not being optimal as it can lead to bias if observations with missing values systematically differ from the complete cases and may inflate standard errors due to the reduction in sample size. Some recent work has been done in this area [96], although it does not consider the case where IPD is available. Finally, another potential issue for future exploration is that the impact of each pain condition on treatment effects was assumed to be exchangeable [82]; this assumption could be explored further by comparing different assumptions over the inclusion of the interaction effects, or even with the inclusion of no interaction effects. In summary, a worthwhile extension to this work would be to develop a multivariate ANCOVA modelling framework considering both multiple endpoints and time points, missing data and which enables relevant aggregate data to be included, building on recent work [15, 97–101].

## Conclusion

In conclusion, this paper has reiterated the importance of accessing and analysing IPD and presented methods to fully exploit the benefits of access to this data in the context of continuous outcomes. Methods for conducting ANCOVA IPD NMA of continuous outcomes are presented and discussed. The methods developed are applicable to contexts in which endpoints are reposted consistently and to contexts in which outcome measures differ across trials. Given the demonstrable benefits of access to IPD, we suggest that more effort should be made to share and develop repositories for data in this format [102].

## Additional files

Additional file 1: Provides summary information of mapped EQ-5D and standardised pain data by study. (DOCX 105 kb)
Additional file 2: Provides WinBUGS modelling code for models 1 and 2 as described in the main text. (DOCX 41 kb)

## Abbreviations

AD: Aggregate data
ANCOVA: Analysis of covariance
ATC: Acupuncture Trialists’ Collaboration
BMI: Body max index
CE: Cost-effectiveness
CMS: Constant Murley Score
CrI: Credibility interval
DIC: Deviance information criterion
EQ-5D: EuroQol five-dimensional
HRQoL: Health-related quality of life
IPD: Individual patient data
MAR: Missing at random
MCMC: Markov chain Monte Carlo
MCS: Mental component summary
NICE: National Institute for Health and Care Excellence
NIHR: National Institute for Health Research
NMA: Network meta-analysis
OAK: Osteoarthritis of the knee
OLS: Ordinary least squares
PCS: Physical component summary
QALY: Quality-adjusted life-year
RD: Residual deviance
SF-12: Short Form 12 dimensions
SF-36: Short Form 36 dimensions
SMD: Standardised mean difference
TRD: Total residual deviance
TTH: Tension-type headache
UK: United Kingdom
VAS: Visual analogue scale
WOMAC: Western Ontario and McMaster Universities Arthritis Index

## Footnotes

1 Considering *s*_*tx*_^*t*^ as the standardised value of the pain measurement *p* made at the time point *t* in patients under treatment *tx*, it can be demonstrated that (*S*_*tx*1_^*t*1^ − *S*_*tx*1_^*t*0^) − (*S*_*tx*0_^*t*1^ − *S*_*tx*0_^*t*0^) = (*S*_*tx*1_^*t*1^ − *S*_*tx*0_^*t*1^) − (*S*_*tx*1_^*t*0^ − *S*_*tx*0_^*t*0^) = *ΔSMD*

2 The selection of the outcome to be mapped was not at random. Preference was given to generic preference-based instruments (i.e. SF-12 and SF-36) and, in its absence, to condition-specific ones (i.e. WOMAC, VAS pain and CMS), conditional on the existence of a valid and published algorithm. WOMAC was used in preference to VAS pain and CMS as it covers a broader definition of HRQoL.

3 A random effects generalised least squares algorithm considering dimensions, dimensions squared and interactions from Rowen et al. [71] was used. A multinomial logit using PCS and MCS summary scores, summary scores squared and interaction terms from Gray et al. [70] was used.

4 An OLS including total WOMAC score, total WOMAC squared, age and gender as covariates from Barton et al. [73] was used. An OLS including VAS pain and VAS pain squared as covariates from Maund et al. [72] was used.

5 A mapping process involves additional sources of uncertainty - the uncertainty in the mapping function regression coefficients and the structure of the mapping model. These additional sources of uncertainty are not accounted for in this analysis.
