# Supplementary material for: Methods for network meta-analysis of continuous outcomes using individual patient data: a case study in acupuncture for chronic pain
Source: BMC Med Res Methodol. 2016 Oct 6;16:131. doi: 10.1186/s12874-016-0224-1 (PMC5053345; doi:10.1186/s12874-016-0224-1)
Supplement: Additional file 1: — Provides summary information of mapped EQ-5D and standardised pain data by study. (DOCX 105 kb) [file 12874_2016_224_MOESM1_ESM.docx]

Additional File 1

Table A1. Mapped EQ-5D data and standardised pain data

| **ID** | **Study 1^st^ author, year** | **Pain group (type)^a^** | **Treatment** | **HRQoL mapped outcome** | **Predicted EQ-5D score:**  **mean (sd)** | | **Pain stand. outcome** | **Standardised pain score:**  **mean (sd)** | |
| --- | --- | --- | --- | --- | --- | --- | --- | --- | --- |
|  |  |  |  |  | *t_0_* - baseline | *t_1_* – 3 months |  | *t_0_* - baseline | *t_1_* – 3 months |
| 1 | Diener 2006 ^[^[^1^](#_ENREF_1)^]^ | H | Usual care | SF-12 | 0.68 (0.268) | 0.759 (0.213) | migraine days | 1.795 (1.142) | 1.222 (1.114) |
|  |  |  | Sham acupuncture |  | 0.696 (0.248) | 0.76 (0.218) |  | 1.629 (0.948) | 1.08 (1.036) |
|  |  |  | Acupuncture |  | 0.683 (0.251) | 0.783 (0.203) |  | 1.705 (0.95) | 0.996 (0.865) |
| 2 | Endres 2007 ^[^[^2^](#_ENREF_2)^]^ | H | Sham acupuncture |  | 0.666 (0.263) | 0.765 (0.237) | tth days | 2.863 (1.065) | 1.258 (1.103) |
|  |  |  | Acupuncture |  | 0.633 (0.281) | 0.757 (0.219) |  | 2.728 (0.931) | 0.943 (0.865) |
| 10 | Haake 2007 ^[^[^3^](#_ENREF_3)^]^ | MSK | Usual care |  | 0.633 (0.216) | 0.648 (0.231) | Von Korff pain intensity score | 4.877 (1.051) | 2.809 (0.944) |
|  |  |  | Sham acupuncture |  | 0.656 (0.208) | 0.666 (0.212) |  | 4.86 (0.946) | 2.485 (1.001) |
|  |  |  | Acupuncture |  | 0.631 (0.236) | 0.668 (0.199) |  | 4.87 (1.002) | 2.325 (0.995) |
| 24 | Scharf 2006 ^[^[^4^](#_ENREF_4)^]^ | OAK | Usual care |  | 0.533 (0.309) | 0.536 (0.325) | WOMAC total score | 3.609 (0.992) | 2.148 (0.999) |
|  |  |  | Sham acupuncture |  | 0.494 (0.34) | 0.593 (0.318) |  | 3.646 (1.021) | 1.697 (0.978) |
|  |  |  | Acupuncture |  | 0.514 (0.323) | 0.596 (0.32) |  | 3.591 (0.986) | 1.566 (0.933) |
| 3 | Jena 2008 ^[^[^5^](#_ENREF_5)^]^ | H | Usual care | SF-36 | 0.725 (0.198) | 0.754 (0.191) | headache days | 1.171 (0.97) | 1.225 (1.033) |
|  |  |  | Acupuncture |  | 0.726 (0.202) | 0.826 (0.159) |  | 1.2 (1.029) | 0.765 (0.911) |
| 4 | Linde 2005 ^[^[^6^](#_ENREF_6)^]^ | H | Usual care |  | 0.741 (0.175) | 0.744 (0.13) | days of moderate to severe pain | 2.082 (1.132) | 1.797 (0.9) |
|  |  |  | Sham acupuncture |  | 0.767 (0.128) | 0.815 (0.133) |  | 1.933 (0.927) | 1.099 (1.018) |
|  |  |  | Acupuncture |  | 0.737 (0.142) | 0.809 (0.138) |  | 2.001 (0.97) | 1.178 (0.963) |
| 5 | Melchart 2005 ^[^[^7^](#_ENREF_7)^]^ | H | Usual care |  | 0.737 (0.164) | 0.721 (0.159) | headache days | 2.534 (1.017) | 1.883 (0.855) |
|  |  |  | Sham acupuncture |  | 0.75 (0.167) | 0.846 (0.126) |  | 2.59 (0.982) | 1.248 (0.954) |
|  |  |  | Acupuncture |  | 0.766 (0.135) | 0.834 (0.144) |  | 2.56 (1.006) | 1.145 (1.001) |
| 6 | Vickers 2004 ^[^[^8^](#_ENREF_8)^]^ | H | Usual care |  | 0.834 (0.148) | 0.806 (0.182) | severity score | 1.732 (1.088) | 1.48 (1.05) |
|  |  |  | Acupuncture |  | 0.789 (0.171) | 0.809 (0.189) |  | 1.593 (0.915) | 1.125 (0.927) |
| 7 | Brinkhaus 2006 ^[^[^9^](#_ENREF_9)^]^ | MSK | Usual care |  | 0.612 (0.185) | 0.635 (0.24) | VAS pain score | 4.714 (0.97) | 1.979 (0.847) |
|  |  |  | Sham acupuncture |  | 0.598 (0.168) | 0.728 (0.187) |  | 4.749 (1.121) | 1.474 (1.005) |
|  |  |  | Acupuncture |  | 0.616 (0.192) | 0.763 (0.196) |  | 4.508 (0.944) | 1.165 (0.963) |
| 17 | Vas 2006 ^[^[^10^](#_ENREF_10)^]^ | MSK | Sham acupuncture |  | 0.556 (0.226) | 0.611 (0.203) | VAS pain score | 4.842 (1.033) | 2.326 (0.724) |
|  |  |  | Acupuncture |  | 0.605 (0.271) | 0.752 (0.215) |  | 4.603 (0.959) | 0.918 (0.697) |
| 20 | Witt 2006 ^[^[^11^](#_ENREF_11)^]^ | MSK | Usual care |  | 0.663 (0.217) | 0.684 (0.22) | Neck pain and disability score | 3.394 (1.004) | 2.698 (0.966) |
|  |  |  | Acupuncture |  | 0.65 (0.209) | 0.769 (0.188) |  | 3.46 (0.995) | 2.079 (0.937) |
| 21 | Witt 2006 ^[^[^12^](#_ENREF_12)^]^ | MSK | Usual care |  | 0.596 (0.232) | 0.638 (0.241) | Hanover functional ability score | -3.027 (0.994) | -3.051 (1.012) |
|  |  |  | Acupuncture |  | 0.585 (0.235) | 0.732 (0.225) |  | -2.957 (1.005) | -3.453 (0.95) |
| 27 | Witt 2005 ^[^[^13^](#_ENREF_13)^]^ | OAK | Usual care |  | 0.564 (0.185) | 0.59 (0.2) | WOMAC total score | 2.675 (1.042) | 2.084 (0.813) |
|  |  |  | Sham acupuncture |  | 0.561 (0.2) | 0.626 (0.218) |  | 2.751 (0.991) | 1.57 (1.001) |
|  |  |  | Acupuncture |  | 0.564 (0.218) | 0.71 (0.19) |  | 2.664 (1.032) | 1.119 (0.921) |
| 28 | Witt 2006 ^[^[^14^](#_ENREF_14)^]^ | OAK | Usual care |  | 0.56 (0.213) | 0.57 (0.225) | WOMAC total score | 2.057 (1.002) | 1.876 (0.921) |
|  |  |  | Acupuncture |  | 0.576 (0.234) | 0.682 (0.234) |  | 2.062 (0.999) | 1.185 (0.956) |
| 8 | Carlsson 2001 ^[^[^15^](#_ENREF_15)^]^ | MSK | Sham acupuncture | VAS pain | 0.604 (0.222) | 0.434 (0.314) | VAS pain score | 2.386 (1.022) | 2.251 (1.154) |
|  |  |  | Acupuncture |  | 0.481 (0.197) | 0.685 (0.246) |  | 2.79 (0.978) | 1.684 (0.911) |
| 9 | Guerra 2004 ^[^[^16^](#_ENREF_16)^]^ | MSK | Sham acupuncture |  | 0.526 (0.229) | 0.632 (0.256) | VAS pain score | 2.857 (0.871) | 1.134 (1.07) |
|  |  |  | Acupuncture |  | 0.525 (0.202) | 0.666 (0.229) |  | 2.732 (1.118) | 0.488 (0.814) |
| 11 | Irnich 2001 ^[^[^17^](#_ENREF_17)^]^ | MSK | Sham acupuncture |  | 0.551 (0.236) | 0.642 (0.259) | VAS pain score | 2.381 (1.113) | 1.527 (1.055) |
|  |  |  | Acupuncture |  | 0.562 (0.225) | 0.636 (0.233) |  | 2.256 (0.913) | 1.277 (0.918) |
| 12 | Kennedy 2008 ^[^[^18^](#_ENREF_18)^]^ | MSK | Sham acupuncture |  | 0.61 (0.25) | 0.653 (0.242) | Roland Morris disability score | 2.382 (1.033) | 1.265 (1.155) |
|  |  |  | Acupuncture |  | 0.568 (0.22) | 0.562 (0.247) |  | 2.359 (0.988) | 0.813 (0.825) |
| 13 | Kerr 2003 ^[^[^19^](#_ENREF_19)^]^ | MSK | Sham acupuncture |  | 0.523 (0.178) | 0.548 (0.204) | VAS pain score | 3.981 (0.924) | 2.324 (1.154) |
|  |  |  | Acupuncture |  | 0.5 (0.132) | 0.514 (0.266) |  | 4.176 (1.065) | 1.943 (0.849) |
| 18 | Vas 2008 ^[^[^20^](#_ENREF_20)^]^ | MSK | Sham acupuncture |  | 0.556 (0.171) | 0.585 (0.254) | CMS | -3.123 (0.971) | -3.346 (0.975) |
|  |  |  | Acupuncture |  | 0.563 (0.155) | 0.623 (0.249) |  | -3.281 (1.026) | -4.102 (0.874) |
| 19 | White 2004 ^[^[^21^](#_ENREF_21)^]^ | MSK | Sham acupuncture |  | 0.548 (0.129) | 0.679 (0.202) | VAS pain score | 3.971 (1.073) | 1.18 (1.039) |
|  |  |  | Acupuncture |  | 0.572 (0.133) | 0.653 (0.228) |  | 3.64 (0.906) | 0.871 (0.943) |
| 22 | Foster 2007 ^[^[^22^](#_ENREF_22)^]^ | OAK | Usual care |  | 0.543 (0.214) | 0.566 (0.279) | WOMAC pain score | 2.463 (1.004) | 1.626 (0.999) |
|  |  |  | Sham acupuncture |  | 0.531 (0.205) | 0.621 (0.234) |  | 2.428 (0.903) | 1.418 (1.029) |
|  |  |  | Acupuncture |  | 0.576 (0.213) | 0.559 (0.266) |  | 2.52 (1.092) | 1.513 (0.969) |
| 25 | Vas 2004 ^[^[^23^](#_ENREF_23)^]^ | OAK | Sham acupuncture | WOMAC total | 0.37 (0.381) | 0.565 (0.352) | WOMAC total score | 3.308 (1.068) | 1.589 (1.037) |
|  |  |  | Acupuncture |  | 0.345 (0.366) | 0.76 (0.233) |  | 3.273 (0.937) | 0.333 (0.452) |
| 26 | Williamson 2007 ^[^[^24^](#_ENREF_24)^]^ | OAK | Usual care |  | 0.424 (0.304) | 0.487 (0.314) | Oxford knee score | 4.886 (1.039) | 5.104 (1.02) |
|  |  |  | Acupuncture |  | 0.405 (0.278) | 0.508 (0.262) |  | 4.841 (0.922) | 4.774 (0.861) |
| 14 | Kleinhenz 1999 ^[^[^25^](#_ENREF_25)^]^ | MSK | Sham acupuncture | CMS and VAS pain | 0.558 (0.127) | 0.577 (0.071) | CMS | -3.986 (1.037) | -3.462 (0.965) |
|  |  |  | Acupuncture |  | 0.566 (0.101) | 0.578 (0.109) |  | -4.483 (0.909) | -4.417 (0.795) |
| 15 | Salter 2006 ^[^[^26^](#_ENREF_26)^]^ | MSK | Usual care | EQ-5D available - no mapping necessary | 0.546 (0.322) | 0.67 (0.254) | Northwick Park pain score | 2.395 (1.156) | 1.518 (0.961) |
|  |  |  | Acupuncture |  | 0.614 (0.268) | 0.734 (0.269) |  | 2.138 (0.73) | 1.342 (1.1) |
| 16 | Thomas 2006 ^[^[^26^](#_ENREF_26)^,^ [^27^](#_ENREF_27)^]^ | MSK | Usual care |  | 0.532 (0.284) | 0.655 (0.274) | SF-36 bodily pain score | -1.815 (1.075) | -2.316 (1.064) |
|  |  |  | Acupuncture |  | 0.534 (0.293) | 0.753 (0.19) |  | -1.839 (0.964) | -2.545 (0.962) |
| 23 | Berman 2004 ^[^[^28^](#_ENREF_28)^]^ | OAK | Usual care |  | 0.65 (0.196) | 0.628 (0.24) | WOMAC pain score | 2.577 (1.059) | 1.851 (1.098) |
|  |  |  | Sham acupuncture |  | 0.615 (0.246) | 0.693 (0.169) |  | 2.547 (0.969) | 1.587 (0.981) |
|  |  |  | Acupuncture |  | 0.608 (0.232) | 0.682 (0.184) |  | 2.552 (0.979) | 1.345 (0.892) |

Legend: a – H = headache, MSK = musculoskeletal, OAK = osteoarthritis of the knee; sd - standard deviation; HRQoL – Health Related Quality of Life; tth – tension type headache; WOMAC - Western Ontario and McMaster Universities Arthritis Index; VAS – Visual Analogue Scale; CMS - Constant Murley Score

**Table A1 references:**

1. Diener HC, Kronfeld K, Boewing G, Lungenhausen M, Maier C, Molsberger A, Tegenthoff M, Trampisch HJ, Zenz M, Meinert R: **Efficacy of acupuncture for the prophylaxis of migraine: a multicentre randomised controlled clinical trial**. *Lancet neurology* 2006, **5**(4):310-316.

2. Endres HG, Bowing G, Diener HC, Lange S, Maier C, Molsberger A, Zenz M, Vickers AJ, Tegenthoff M: **Acupuncture for tension-type headache: a multicentre, sham-controlled, patient-and observer-blinded, randomised trial**. *The journal of headache and pain* 2007, **8**(5):306-314.

3. Haake M, Muller HH, Schade-Brittinger C, Basler HD, Schafer H, Maier C, Endres HG, Trampisch HJ, Molsberger A: **German Acupuncture Trials (GERAC) for chronic low back pain: randomized, multicenter, blinded, parallel-group trial with 3 groups**. *Archives of internal medicine* 2007, **167**(17):1892-1898.

4. Scharf HP, Mansmann U, Streitberger K, Witte S, Kramer J, Maier C, Trampisch HJ, Victor N: **Acupuncture and knee osteoarthritis: a three-armed randomized trial**. *Annals of internal medicine* 2006, **145**(1):12-20.

5. Jena S, Witt CM, Brinkhaus B, Wegscheider K, Willich SN: **Acupuncture in patients with headache**. *Cephalalgia : an international journal of headache* 2008, **28**(9):969-979.

6. Linde K, Streng A, Jurgens S, Hoppe A, Brinkhaus B, Witt C, Wagenpfeil S, Pfaffenrath V, Hammes MG, Weidenhammer W *et al*: **Acupuncture for patients with migraine: a randomized controlled trial**. *JAMA : the journal of the American Medical Association* 2005, **293**(17):2118-2125.

7. Melchart D, Streng A, Hoppe A, Brinkhaus B, Witt C, Wagenpfeil S, Pfaffenrath V, Hammes M, Hummelsberger J, Irnich D *et al*: **Acupuncture in patients with tension-type headache: randomised controlled trial**. *Bmj* 2005, **331**(7513):376-382.

8. Vickers AJ, Rees RW, Zollman CE, McCarney R, Smith CM, Ellis N, Fisher P, Van Haselen R, Wonderling D, Grieve R: **Acupuncture of chronic headache disorders in primary care: randomised controlled trial and economic analysis**. *Health Technol Assess* 2004, **8**(48):iii, 1-35.

9. Brinkhaus B, Witt CM, Jena S, Linde K, Streng A, Wagenpfeil S, Irnich D, Walther HU, Melchart D, Willich SN: **Acupuncture in patients with chronic low back pain: a randomized controlled trial**. *Archives of internal medicine* 2006, **166**(4):450-457.

10. Vas J, Perea-Milla E, Mendez C, Sanchez Navarro C, Leon Rubio JM, Brioso M, Garcia Obrero I: **Efficacy and safety of acupuncture for chronic uncomplicated neck pain: a randomised controlled study**. *Pain* 2006, **126**(1-3):245-255.

11. Witt CM, Jena S, Brinkhaus B, Liecker B, Wegscheider K, Willich SN: **Acupuncture for patients with chronic neck pain**. *Pain* 2006, **125**(1-2):98-106.

12. Witt CM, Jena S, Selim D, Brinkhaus B, Reinhold T, Wruck K, Liecker B, Linde K, Wegscheider K, Willich SN: **Pragmatic randomized trial evaluating the clinical and economic effectiveness of acupuncture for chronic low back pain**. *American journal of epidemiology* 2006, **164**(5):487-496.

13. Witt C, Brinkhaus B, Jena S, Linde K, Streng A, Wagenpfeil S, Hummelsberger J, Walther HU, Melchart D, Willich SN: **Acupuncture in patients with osteoarthritis of the knee: a randomised trial**. *Lancet* 2005, **366**(9480):136-143.

14. Witt CM, Jena S, Brinkhaus B, Liecker B, Wegscheider K, Willich SN: **Acupuncture in patients with osteoarthritis of the knee or hip: a randomized, controlled trial with an additional nonrandomized arm**. *Arthritis and rheumatism* 2006, **54**(11):3485-3493.

15. Carlsson CP, Sjolund BH: **Acupuncture for chronic low back pain: a randomized placebo-controlled study with long-term follow-up**. *The Clinical journal of pain* 2001, **17**(4):296-305.

16. Guerra de Hoyos JA, Andres Martin Mdel C, Bassas y Baena de Leon E, Vigara Lopez M, Molina Lopez T, Verdugo Morilla FA, Gonzalez Moreno MJ: **Randomised trial of long term effect of acupuncture for shoulder pain**. *Pain* 2004, **112**(3):289-298.

17. Irnich D, Behrens N, Molzen H, Konig A, Gleditsch J, Krauss M, Natalis M, Senn E, Beyer A, Schops P: **Randomised trial of acupuncture compared with conventional massage and "sham" laser acupuncture for treatment of chronic neck pain**. *Bmj* 2001, **322**(7302):1574-1578.

18. Kennedy S, Baxter GD, Kerr DP, Bradbury I, Park J, McDonough SM: **Acupuncture for acute non-specific low back pain: a pilot randomised non-penetrating sham controlled trial**. *Complementary therapies in medicine* 2008, **16**(3):139-146.

19. Kerr DP, Walsh DM, Baxter D: **Acupuncture in the management of chronic low back pain: a blinded randomized controlled trial**. *The Clinical journal of pain* 2003, **19**(6):364-370.

20. Vas J, Ortega C, Olmo V, Perez-Fernandez F, Hernandez L, Medina I, Seminario JM, Herrera A, Luna F, Perea-Milla E *et al*: **Single-point acupuncture and physiotherapy for the treatment of painful shoulder: a multicentre randomized controlled trial**. *Rheumatology (Oxford)* 2008, **47**(6):887-893.

21. White P, Lewith G, Prescott P, Conway J: **Acupuncture versus placebo for the treatment of chronic mechanical neck pain: a randomized, controlled trial**. *Annals of internal medicine* 2004, **141**(12):911-919.

22. Foster NE, Thomas E, Barlas P, Hill JC, Young J, Mason E, Hay EM: **Acupuncture as an adjunct to exercise based physiotherapy for osteoarthritis of the knee: randomised controlled trial**. *Bmj* 2007, **335**(7617):436.

23. Vas J, Mendez C, Perea-Milla E, Vega E, Panadero MD, Leon JM, Borge MA, Gaspar O, Sanchez-Rodriguez F, Aguilar I *et al*: **Acupuncture as a complementary therapy to the pharmacological treatment of osteoarthritis of the knee: randomised controlled trial**. *Bmj* 2004, **329**(7476):1216.

24. Williamson L, Wyatt MR, Yein K, Melton JT: **Severe knee osteoarthritis: a randomized controlled trial of acupuncture, physiotherapy (supervised exercise) and standard management for patients awaiting knee replacement**. *Rheumatology (Oxford)* 2007, **46**(9):1445-1449.

25. Kleinhenz J, Streitberger K, Windeler J, Gussbacher A, Mavridis G, Martin E: **Randomised clinical trial comparing the effects of acupuncture and a newly designed placebo needle in rotator cuff tendinitis**. *Pain* 1999, **83**(2):235-241.

26. Salter GC, Roman M, Bland MJ, MacPherson H: **Acupuncture for chronic neck pain: a pilot for a randomised controlled trial**. *BMC Musculoskelet Disord* 2006, **7**:99.

27. Thomas KJ, MacPherson H, Ratcliffe J, Thorpe L, Brazier J, Campbell M, Fitter M, Roman M, Walters S, Nicholl JP: **Longer term clinical and economic benefits of offering acupuncture care to patients with chronic low back pain**. *Health Technol Assess* 2005, **9**(32):Iii-+.

28. Berman BM, Lao L, Langenberg P, Lee WL, Gilpin AM, Hochberg MC: **Effectiveness of acupuncture as adjunctive therapy in osteoarthritis of the knee: a randomized, controlled trial**. *Annals of internal medicine* 2004, **141**(12):901-910.
