# Supplementary material for: Methods for network meta-analysis of continuous outcomes using individual patient data: a case study in acupuncture for chronic pain
Source: BMC Med Res Methodol. 2016 Oct 6;16:131. doi: 10.1186/s12874-016-0224-1 (PMC5053345; doi:10.1186/s12874-016-0224-1)
Supplement: Additional file 2: — Provides WinBUGS modelling code for models 1 and 2 as described in the main text. (DOCX 41 kb) [file 12874_2016_224_MOESM2_ESM.docx]

**Additional File 2**

**WinBUGS code (model 1 and model 2)**

The WinBUGS modelling code is provided below followed by a summary table of all variables included in the dataset and R-code describing the specification of initial values for two chains.

**WinBUGS model code**

model {

sw[1]<- 0

for(i in 1:n.subjects) {

#likelihood function

y[i] ~ dnorm(theta[i], prec[study[i]])

theta[i] <- mu[study[i]] + delta[index[i]] * (1 - equals(treat[i],baseline[i])) +

b_basey * bl_y[i]

+ b_treat_pain1[index[i]] * (1 - equals(treat[i],baseline[i])) * pain1[i] + #exclude if not interested in pain type interactions

b_treat_pain2[index[i]] * (1 - equals(treat[i],baseline[i])) * pain2[i] #exclude if not interested in pain type interactions

#residual deviance

dev[i]<- (y[i] - theta[i]) * (y[i] - theta[i])

}

for(l in 1:n.arms) {

#consistency model for treatment effects and pain type interactions

delta[l]~dnorm(md[l], precd[l])

md[l]<- d[treat1[l]] - d[baseline1[l]] + equals(m[l],3) * sw[l]

precd[l]<- pre * (1 + equals(m[l],3) / 3)

b_treat_pain1[l] <- btp1[treat1[l]] - btp1[baseline1[l]] #exclude if not interested in pain type interactions

b_treat_pain2[l] <- btp2[treat1[l]] - btp2[baseline1[l]] #exclude if not interested in pain type interactions

}

#correction for multi-arm trials

for(k in 2:n.arms) {

sw[k]<- (delta[k-1] - d[treat1[k-1]] + d[baseline1[k-1]]) / 2

}

for(j in 1:n.trials) {

#priors on study-specific baseline outcome and precision parameter

mu[j]~dnorm(0,1.0E-6)

prec[j] ~ dgamma(0.001, 0.001)

# summed residual deviance contribution for each trial

resdev[j]<- sum(dev[start[j]:end[j]]) * prec[j]

}

#total Residual Deviance

totresdev <- sum(resdev[])

for (k in 2:NT) {

#random effect on pain interaction effects #exclude if not interested in pain type interactions

btp1[k] ~ dnorm(m.btp1,prec.btp1) #exclude if not interested in pain type interactions

btp2[k] ~ dnorm(m.btp2,prec.btp2) #exclude if not interested in pain type interactions

#prior on treatment effects

d[k] ~ dnorm(0,1.0E-6)

}

#prior on random treatment effect variance

tau ~ dunif(0,10)

tau.sq <- tau * tau

pre<- 1 / (tau.sq)

#priors on mean and random effect variance for pain type interaction #exclude if not interested in pain type interactions

m.btp1 ~ dnorm(0,1.0E-6) #exclude if not interested in pain type interactions

tau.btp1 ~ dunif(0,2) #exclude if not interested in pain type interactions

tau.sq.btp1 <- tau.btp1 * tau.btp1 #exclude if not interested in pain type interactions

prec.btp1 <- 1 / (tau.sq.btp1) #exclude if not interested in pain type interactions

m.btp2 ~ dnorm(0,1.0E-6) #exclude if not interested in pain type interactions

tau.btp2 ~ dunif(0,2) #exclude if not interested in pain type interactions

tau.sq.btp2 <- tau.btp2 * tau.btp2 #exclude if not interested in pain type interactions

prec.btp2 <- 1 / (tau.sq.btp2) #exclude if not interested in pain type interactions

#prior on impact of baseline outcome on final outcome

b_basey ~ dnorm(0,1.0E-6)

d[1]<-0

btp1[1]<-0

btp2[1]<-0

}

**Data**

**Table: Description of datasets and variables**

| Data set | Variable | Description |
| --- | --- | --- |
| Data set descriptors, constants | n.subjects | Number of subjects in dataset |
|  | n.arms | Number of arms in studies in dataset |
|  | n.trials | Number of trials in dataset |
|  | NT | Number of treatments in dataset |
| Data at individual patient level | study | Study code |
|  | treat | Treatment code |
|  | baseline | Baseline (lowest index) treatment in study |
|  | y | EQ-5D or standardised pain at follow-up |
|  | bl_y | EQ-5D or standardised pain at baseline |
|  | index | Sequential numbering of study arms in dataset |
| Data at study arm level | pain1 | Dummy variable indicating whether study was conducted in patients with headache pain |
|  | pain2 | Dummy variable indicating whether study was conducted in patients with musculoskeletal pain |
|  | treat1 | Treatment code |
|  | baseline1 | Baseline (lowest index) treatment in study |
|  | m | Code for each study arm indicating whether it is the 1st, 2nd, 3rd, 4th, 5th arm in the study |
| Data at study level | start | Number of the first line of IPD dataset including study data |
|  | end | Number of the last line of IPD dataset including study data |

**R-code used to generate initial values (two sets)**

list(list(d = c(NA,0,0), mu = c(0,0,0,0,0, 0,0,0,0,0, 0,0,0,0,0, 0,0,0,0,0, 0,0,0,0,0, 0,0,0), delta = c(0,0,0,0,0, 0,0,0,0,0, 0,0,0,0,0, 0,0,0,0,0, 0,0,0,0,0, 0,0,0,0,0, 0,0,0,0,0, 0,0,0,0,0, 0,0,0,0,0, 0,0,0,0,0, 0,0,0,0,0, 0,0,0,0,0, 0,0,0,0,0), b_basey=c(0), tau = c(1), btp1 = c(NA,0,0), btp2 = c(NA,0,0), prec=c(1,1,1,1,1, 1,1,1,1,1, 1,1,1,1,1, 1,1,1,1,1, 1,1,1,1,1, 1,1,1), tau.btp1 = c(1), tau.btp2 = c(1)), list(d = c(NA,-1,1), mu = c(-3,-3,-3,3,3, -3,-3,-3,3,3, -3,-3,-3,3,3, -3,-3,-3,3,3, -3,-3,-3,3,3, -3,-3,-3), delta = c(0,0,0,0,0, 0,0,0,0,0, 0,0,0,0,0, 0,0,0,0,0, 0,0,0,0,0, 0,0,0,0,0, 0,0,0,0,0, 0,0,0,0,0, 0,0,0,0,0, 0,0,0,0,0, 0,0,0,0,0, 0,0,0,0,0, 0,0,0,0,0), b_basey=c(1), tau = c(1), btp1 = c(NA,0,0), btp2 = c(NA,0,0), prec=c(1,1,1,1,1, 1,1,1,1,1, 1,1,1,1,1, 1,1,1,1,1, 1,1,1,1,1, 1,1,1), tau.btp1 = c(1), tau.btp2 = c(1)))
